# Disease-Modifying Therapies for Alzheimer’s Disease: More Questions than Answers

**DOI:** 10.1007/s13311-022-01201-2

**Published:** 2022-02-28

**Authors:** Todd E. Golde

**Affiliations:** Departments of Neuroscience and Neurology, Center for Translational Research in Neurodegenerative Disease, Evelyn F. and William L. McKnight Brain Institute, Norman Fixel Institute for Neurological Diseases, University of Florida, Gainesville, FL USA

**Keywords:** Alzheimer’s disease, Therapeutics, Amyloid, Tau, Inflammation, Prevention, Disease modification

## Abstract

Scientific advances over the last four decades have steadily infused the Alzheimer’s disease (AD) field with great optimism that therapies targeting Aβ, amyloid, tau, and innate immune activation states in the brain would provide disease modification. Unfortunately, this optimistic scenario has not yet played out. Though a recent approval of the anti-Aβ aggregate binding antibody, Aduhelm (aducanumab), as a “disease-modifying therapy for AD” is viewed by some as a breakthrough, many remain unconvinced by the data underlying this approval. Collectively, we have not succeeded in changing AD from a largely untreatable, inevitable, and incurable disease to a treatable, preventable, and curable one. Here, I will review the major foci of the AD “disease-modifying” therapeutic pipeline and some of the “open questions” that remain in terms of these therapeutic approaches. I will conclude the review by discussing how we, as a field, might adjust our approach, learning from our past failures to ensure future success.

**Supplementary Information:**

The online version contains supplementary material available at 10.1007/s13311-022-01201-2.

## Introduction

AD is the most common form of dementia, accounting for ~ 70% of all dementias in those over 60. Worldwide, it is predicted that the estimated 35–40 million individuals currently affected by AD will grow to at least 100 million individuals with AD in 2050 [1]. AD prevalence varies depending on the population studied and criteria used to define the disease, but prevalence rises with increasing age [1, 2]. Thus, with increased life span comes an increased risk for developing AD. Despite the recent Food and Drug Administration (FDA) approval of Aduhelm (aducanumab), hailed by some as the first disease-modifying therapy for AD, there remains a great unmet medical need [3]. Indeed, even if one takes the most optimistic “cherry-picked” view of the Aduhelm data from the phase 3 trials, the average clinical impact in terms of slowing cognitive decline is quite modest [4, 5].

Recent reviews have catalogued the therapeutic AD pipeline [6–8]. Rather than rehash these scholarly and detailed manuscripts, I will focus this review on three major therapeutic categories, Aβ-, tau-, and immune-targeting therapies, that are being developed for AD and related disorders. Within each section, I will begin with a high-level overview of the scientific rationale supporting the therapeutic approach, follow this with a concise summary of pivotal human clinical trial data, if such data is available, then address a series of “open questions” that reflect critical knowledge gaps regarding that form of therapy. The review will thus focus on small molecule and genetic and biologic therapies, which are designed to be disease modifying by targeting some aspect of the pathophysiology of AD. I will discuss the need for reinvestment in development of symptomatic therapies at the end of this review, but will not critically review the ongoing efforts to develop novel symptomatic therapies.

## Aβ- and Amyloid-Targeting Therapies

### Overall Rationale

Genetic, human biomarker, pathologic, and experimental modeling studies strongly support the hypothesis that Aβ aggregate accumulation in the brain is the triggering causal event in AD [9]. Though there remain many gaps in our understanding of how the slow accumulation of Aβ aggregates triggers the complex downstream cellular pathophysiology that are characteristic of the symptomatic phase of AD, targeting Aβ with the goal of altering amyloid deposition has been the mainstay of AD therapeutic development [10, 11].

### Immunotherapies Targeting Aβ in AD

#### Rationale

Disruptive studies, initially conducted by Shenk and colleagues during the late 1990s and early 2000s, demonstrated both active and passive immunotherapies targeting Aβ could reduce amyloid accumulation in preclinical mouse models of Aβ amyloid deposition [10, 11]. These preclinical studies showing reduction of amyloid by Aβ immunotherapies have now been reproduced in hundreds of independent preclinical studies (reviewed in [12, 13]). The studies were disruptive as they overturned dogma that the limited central nervous system (CNS) exposure of an antibody therapeutic would render this approach ineffective. Indeed, multiple studies show that ~ 0.1% of a peripheral antibody crosses the blood–brain barrier, limiting central nervous system (CNS) exposure [12, 14, 15]. This limited CNS exposure has many implications for anti-Aβ therapy that we now understand are important for development and selection of the most efficacious anti-Aβ (at least with respect to CNS targeting of amyloid). Multiple possible mechanisms for antibody mediated reductions and clearance of Aβ have been proposed and supported by various preclinical studies (reviewed in [12, 16, 17]). How human studies have informed these preclinic mechanistic studies will be discussed below.

### Active Vaccination Targeting Aβ: AN1792

#### Clinical Studies

Initial studies reported on the efficacy of an active vaccination with fibrillar Aβ42 reducing amyloid deposition in mice with no apparent ill-effects [18], rapidly led to the first human trial of an anti-Aβ vaccine, AN-1792. This vaccine was fibrillar human Aβ42 in conjunction with a novel adjuvant QS-21. Clinical studies of AN-1792 were halted during phase 2 due to a 6% rate of aseptic meningoencephalitis with only an ~ 20% rate of “adequate” humoral responses to Aβ [19, 20]. Over time, a number of studies have emerged from follow-up of those enrolled in the trials [21–25]. These studies show possible hints of “amyloid plaque reduction” in postmortem tissue and possible functional benefit in responders [21, 23, 25, 26]. T-cell infiltration was described in the autopsy from the patient with meningoencephalitis [26]. Brain volume reductions were also noted based on structural MRI. Non-systematic analysis of postmortem brains collected from long-term follow provides suggestive support that in some individuals, there were regional reductions in amyloid loads [23, 25].

#### What Was Learned from This Study?

The top-line lessons are that (i) immunizing humans with a self-antigen and novel adjuvant warrants a great deal of caution as there is a reasonable potential for unexpected and unwanted immune responses that are not observed in preclinic studies and [2] in elderly humans, it is not easy to generate a robust humoral response to a self-antigen. Furthermore, the study highlighted the need for better biomarkers of AD to [1] ensure trial participants truly had AD and [2] to assess target engagement and efficacy in effective targeting of the pathology. Such biomarkers were just beginning to emerge near the end of this trial and, therefore, are not included in the trial design.

### Open Questions from the AN-1792 Study

1. *What caused the meningoencephalitis?* The severe meningoencephalitis observed in some individuals in the phase 2 trial in many cases responded to steroid treatment suggesting that it was likely a T-cell mediated response, a finding consistent with post hoc postmortem studies and analysis of mRNA transcripts in the blood [21, 26]; however, as peripheral cellular immune responses were not monitored during the trial, definitive data that the meningoencephalitis was attributable to a T-cell response and more specially a T-cell response to Aβ is lacking. Nevertheless, the concept that this was a T-cell response to Aβ, and in particular to a T-cell epitope within the carboxyl-terminus of Aβ, guided future vaccine development.
2. *Did the use of QS-21 contribute to the unexpected side effects observed?* The saponin QS-21 is one of the active fractions of the bark of Chilean tree, *Quillaja saponaria*, and is an acylated 3, 28-bisdesmodic triterpene glycoside [27]. It is one of the most potent immunological adjuvants that has been used in humans and dose in most patient populations is limited by toxicities. Indeed, it has only recently been approved for human use as one of a multi-component adjuvant in a few vaccines [28, 29]. Given its potent immune stimulating nature, the contribution of this adjuvant to the side effects is unclear, but should not be discounted.
3. *Was there really amyloid reduction and, if so, what response to the vaccine caused the reduction?* PET amyloid tracers were not available at the time of the AN-1792 trial. Thus, post hoc postmortem studies claiming “clearance” of amyloid plaques rely on a number of circumstantial findings to claim actual amyloid reduction. The original case report on an individual who died from the meningoencephalitis was intriguing in that the “clearance” was very circumscribed within select regions of the brain [26]. These “patchy” effects are reminiscent of T-cell-mediated CNS disease that occur in disorders like multiple sclerosis. Additional studies on larger cohorts of AN-1792 vaccinated individuals also claim reductions in amount of amyloid deposited in some areas of the brain, relative to what might be expected [23, 25]; however, this data is again circumstantial. Without definitive data showing amyloid status prior to receiving the drug, it cannot be proven amyloid was there and then subsequently reduced. Certainly, the data is provocative. Though a correlation between anti-Aβ antibody titers and extent of the claimed amyloid reductions is shown, several individuals with little amyloid at autopsy showed no, or almost no, anti-Aβ titer. This data could be explained in a number of ways. At face value, such data would suggest that the effects on amyloid in these trials were not due to a humoral response to Aβ, but either a non-specific T-cell or an Aβ/amyloid targeting T-cell response. Alternately, but less likely, would be that titering protocol missed some type of conformational antibody response to Aβ that mediated the efficacy. Finally, it is also quite possible these individuals really did not have AD at the time the trial was initiated, so that there was little Aβ to be cleared.
4. *What is a sufficient titer for an active vaccine and to what form of Aβ?* Titering of antibodies simply based on dilution is not a particularly precise method to determine the response to a given vaccine. Such titering allows for one to rank order responses among individuals, but does not allow comparison across trials. An anti-Aβ titer converted to actual molar concertation of the antibody against Aβ would be much more useful, but even then, standardization of the Aβ peptides and assays used to assess the titer would be needed to benchmark and standardize across trials. As discussed below, both preclinical and clinical studies with monoclonal anti-Aβ antibodies demonstrate that epitope, selectivity for aggregates, affinity, and, to some degree, effector functions appear to be important aspects of a given monoclonal antibody’s ability to impact amyloid deposition [17]. Whether any vaccine targeting Aβ can generate a humoral response with a preponderance of antibodies of appropriate affinity and specificity remains unclear.

### Second-generation Active Vaccinations Targeting Aβ

#### Clinical Studies

A number of additional active vaccine candidates targeting Aβ have been developed and moved into clinical trials. A continuously updated summary of these and other classes of AD therapeutics can be found on AlzForum (https://www.alzforum.org/therapeutics). Development of several of these vaccines has been discontinued or is inactive, whereas three vaccines (AbVac40, phase 2; ACI-24, phase 2; and UBI-311, phase 3) remain in later phase clinical trials [30–34]. Building off data gleaned from the AN1792 trial, these vaccines have generally been developed using a restricted B-cell epitope of Aβ and some alternative helper T-cell epitope. Many are claimed to produce robust anti-Aβ humoral responses, but as noted above, the lack of benchmarking and standardization of such titers prevents a truly rigorous assessment of the adequacy of response.

### Open Questions for Second-generation Vaccines Targeting Aβ

1. *Safety looks more promising, but is the humoral response against Aβ sufficient to impact disease?* Available data suggest that most, if not all, of these second-generation vaccines avoid the meningoencephalitis induced by AN1792. Some also seem to generate more consistent humoral immune responses to Aβ; however, whether the titers and the quality of the antibody responses to Aβ are sufficient to engage Aβ in the brain and, more particularly Aβ aggregates, is simply not known [30–34].
2. *What can we infer from the lack of data on amyloid PET ligand reduction?* As opposed to data for select monoclonal antibodies, there is no published data available that demonstrates reduction, or slowing of the increase, in amyloid PET ligand signal in the brain following treatment with any active vaccine targeting Aβ. Such data, or data showing some major impact on a biomarker of neurodegeneration, would certainly reignite enthusiasm for an active vaccination approach targeting Aβ.
3. *At least with respect to amyloid reduction, could the T-cell to Aβ response be important?* Based on the AN1792 trial, a T-cell response to Aβ has been hypothesized to be something to be avoided [26]; however, this hypothesis was not tested in the AN1792 trial, nor has it ever been fully evaluated in preclinic models. Generation of chimeric antigen receptor (CAR) T-cells targeting Aβ and testing of these in animal models of Aβ deposition might provide insight into the role that T-cells might play in active vaccination paradigms [35]. Indeed, the assertion that the meningoencephalitis observed in the AN1792 trial was an autoreactive T-cell response to Aβ could be formally tested in such studies. Alternately, such an approach could show unexpected efficacy, as it is well established that T-cells survey the brain and play a role in regulating immune activation states in the CNS.

### Passive Immunotherapy Targeting Aβ

#### Rationale

Numerous preclinical studies have shown the ability of peripherally administered antibodies targeting Aβ to attenuate amyloid deposition in the brains of amyloid depositing mice (reviewed in [12, 16, 17]). Behavioral improvements have also been noted in these mice following select antibody administration. It is important to note that these preclinical studies reproducibly show clearance of diffuse amyloid deposit, but typically show minimal impact on preexisting amyloid cores. “Clearance” is often loosely used to describe the impact of immunotherapies and the field should be cautious about claims of amyloid clearance as opposed to slowing or attenuating additional deposition. Some side effects, including microhemorrhages, have been noted in select preclinical studies as well.

A large number of passive immunotherapies targeting Aβ have been advanced to human studies. As mentioned previously, one of these, aducanumab (Aduhelm), is now approved for use, though as noted, the approval and supporting data remain controversial [4, 5].

#### Clinical Studies: What Has Been Learned?

Given the large number of antibodies tested to date, a picture of the properties that are associated with potential efficacy is emerging. These data reveal that antibodies that selectively bind to deposited Aβ, either because they bind aggregates with higher selectivity than monomer (Aduhelm [36], lecanemab [37], and gantenerumab [38, 39]) or bind modified forms of Aβ such as N3-pGAβ (donanemab[40–42]) highly enriched in deposited Aβ, can effectively lower amyloid PET ligand signals to control, or near control, levels in many individuals with early clinical stages of AD. There is data that has hinted at slowing of cognitive decline in almost all the early phase trials of these antibodies and, as noted above, highly controversial data of possible cognitive and functional improvement in the phase 3 trials for Aduhelm. Another common feature of these antibodies is that they contain activating Fc domains and to some degree induce radiographic features known as Alzheimer’s related imaging abnormalities with edema (ARIA-E) or hemorrhage (ARIA-H) [43]. Both types of ARIA can be associated with some cognitive side effects that are typically mild and that also typically resolve upon discontinuation or lowering of dose. Because of this association, it is thought that ARIA is, in fact, a marker of target engagement.

A number of antibodies that have advanced to phase 3 studies in symptomatic AD have not shown evidence for efficacy either with respect to marked lowering of the PET ligand signal, or any significant clinical impact (ponezumab [44], solanezumab [39, 45–47], bapineuzumab [48], crenezumab [49]). Commonalties among these antibodies are that they do not show much differential binding between Aβ monomers and Aβ aggregates, or preferentially bind only Aβ monomers, and, in some cases, were designed to minimize the immune activation by limiting Fc engagement of activating immune receptors.

Overall, the studies conducted to date are consistent with a model whereby an anti-Aβ antibody must enter the brain and engage deposited Aβ [17]. Subsequently, the presence of the antibody with an activating Fc domain is thought to elicit a microglial response that then reduces deposited Aβ to a degree that results in a reduction in amyloid PET ligand signals. Binding to soluble monomer with high affinity is a liability, in sense blunting the amount of antibody that can bind to deposits in the brain.

### Open Questions for Passive Aβ Immunotherapies

1. *Is the clinical efficacy in symptomatic disease sufficient to warrant use?* Though Aduhelm has now been approved by the FDA, it is clear that many, if not most, in the field remain unconvinced of the clinical benefits of this antibody therapy [4, 5]. Indeed, both the European Medical Agency’s decision to decline marketing approval for Aduhelm and the recent Centers for Medicare and Medicaid Services (CMS) draft decision that may limit Aduhelm coverage to clinical trials (https://www.alzforum.org/news/research-news/cms-plans-limit-aduhelm-coverage-clinical-trials) reflect the concerns over the FDA approval.
  Despite clear evidence for reduction in amyloid PET ligand signal in the brain, effects on slowing cognitive decline and improvement of function (e.g., activities of daily living) remain uncertain. Even the most optimistic take on the Aduhelm data would suggest that an ~ 20% slowing of cognitive decline might result from this treatment. Given that high-dose Aduhelm treatment results in a large increase in ARIA (41%) compared to placebo (10%), with ~ 1% of those treated with Aduhelm showing serious side effects, the high cost of the drug, and the intensive treatment regiment, it is clear that a larger clinical signal is needed to convince many stakeholders that this drug should become standard of care [4, 5, 50]. The other monoclonal antibodies (lecanemab, gantenerumab, and donanemab) with the ability to lower amyloid PET ligand signal are in late phase clinical testing. We can only hope that data from these trials will reveal a more consistent picture with respect to clinical efficacy.
2. *Are these antibodies really clearing amyloid?* It is clear that in many individuals receiving select Aβ aggregate targeting monoclonal antibodies that treatment overtime reduces amyloid PET ligand signal in the brain. Indeed, the FDA cited this biomarker impact as a major reason for approval; however, we still lack critical confirmation that the reduction in amyloid PET ligand signal equates to a reduction in deposited Aβ. Unequivocal demonstration of amyloid clearance in a postmortem brain is needed to establish this relationship and we simply do not have such data. Though amyloid PET signal correlates very well with amyloid deposition [51], it is clear that it is not a truly selective biomarker of cored plaque pathology, as areas of the brain with only diffuse amyloid can show marked amyloid PET ligand binding [52, 53]. Furthermore, preclinical studies show ability of many antibodies to alter diffuse Aβ deposited, but cored plaque pathology is highly resistant to clearance [17]. It would seem that if the field and regulatory bodies are going to use amyloid ligand binding as a biomarker, then we should go the distance and really establish the relationship between amyloid PET ligand reduction and impact on amyloid depositon in the brain through postmortem pathological studies.
3. *Is it possible to reduce ARIA and still have meaningful impact on the amyloid PET-ligand signal?* Monoclonal antibody therapies that result in reductions of amyloid PET ligand signal all appear to induce some degree of ARIA. Incidence of ARIA is increased in those with evidence for preexisting vascular amyloid based on presence of preexisting microhemorrhage and also in individuals with E4 alleles, who typically have more cerebrovascular amyloid deposition [36–43, 50]. Higher doses of antibody also result in a higher incidence of ARIA. These data suggest that effector functions of these antibodies and binding to cerebrovascular amyloid may underlie this sometimes dose-limiting impact of Aβ monoclonal antibody therapy. Though most cases of ARIA are mild and spontaneously resolve, serious adverse outcomes are observed in some who develop ARIA.
  There are many theories about the biological basis of ARIA, but we have little direct data that provides mechanistic insight into this effect of anti-Aβ antibody administration. Spontaneous ARIA-like events are observed in AD and have been linked to the presence of cerebrovascular amyloid angiopathy (CAA) and the presence of auto-antibodies against Aβ [54]. Given the current data, it is certainly plausible to hypothesize that engagement of cerebrovascular amyloid by an antibody with immune activating effector functions results in a local immune response that results in radiographic changes, and edema, that are consistent with local inflammatory responses. This response could remove amyloid from the vessels and subsequently increase cortical hemorrhages. It is also possible that engagement of deposited Aβ by an antibody in the parenchyma can also contribute to ARIA.
  Though most cases of ARIA are not severe, the relatively common induction of ARIA certainly complicates management of patients undergoing treatment with antibodies that reduce the amyloid PET ligand signal. Furthermore, in some individuals, ARIA represents a severe side effect and negatively impacts the risk benefit equation. This raises an important question about whether it will be possible to either develop anti-Aβ antibodies that are capable of reducing the amyloid PET ligand signal without inducing ARIA, or if dosing paradigms or timing of treatment could be altered to reduce it. Indeed, it has been reported that risk for ARIA diminishes as treatment progresses. Again, lack of study of postmortem tissue from individuals who developed ARIA limits both our mechanistic understanding of it and future modifications to immunotherapies that might limit its impact.
4. *Have these studies truly tested the amyloid hypothesis?* The simple answer to this question is no. The amyloid hypothesis posits that Aβ accumulation in the brain triggers a complex neurodegenertive cascade that over decades leads to a state akin to brain organ failure [9]. Simply put, the field has spent a long-time testing a pretty low probability event — that targeting of amyloid in the symptomatic phase of disease will have a major impact on disease [10]. Though there may yet be clinical benefit that emerges from ongoing symptomatic trials of anti-Aβ immunotherapies, the data that we have in hand would suggest a limited impact of these agents in the symptomatic stage of disease. Indeed, I and others have previously discussed that the only true test of the amyloid hypothesis is to prevent amyloid deposition in humans and see if that prevents the development of AD [10, 55]. Unfortunately, the antibodies that appear capable of robust engagement of deposited Aβ have not yet been rigorously studied in either primary or secondary prevention studies.

### Aβ Production and Aggregation Inhibitors

#### Rationale

There is no evidence that monomeric Aβ is pathogenic and there is little convincing evidence that it has a normal necessary physiologic function [56]; however, it is clear that upon aggregation, accumulation, and deposition, Aβ aggregates can have a plethora of pathophysiologic functions [57, 58]. Given that Aβ aggregation is concentration-dependent phenomenon, lowering levels of Aβ can reduce, slow, or even completely block aggregation. Aβ is normally produced through the sequential actions of the β-and γ-secretases on the amyloid β protein precursor (APP) and inhibitors that target these proteases have been developed and shown to block production of all species of Aβ in the brain [56]. Furthermore, it has been shown that longer Aβ species (primarily Aβ42, but in some cases Aβ43), which are produced at lower levels than the predominant Aβ40 species, are required for in vivo aggregation of Aβ [59–62]. Thus, selective targeting of these species using modulators of γ-secretase activity (GSMS) has also emerged as a possible way to target Aβ production and subsequently alter deposition [63, 64].

Given that Aβ aggregates, and not monomer, are pathogenic, many efforts have been made to develop inhibitors of Aβ aggregation [65–71]. Though many of these have shown ability to block Aβ aggregation in vitro, data in preclinical animal models of amyloid deposition has always been much less impressive. Nevertheless, several anti-Aβ aggregation agents remain in clinical development including ALZ-801 [72] and PRI-002 [73].

#### Clinical Studies

The first Aβ production inhibitors to be tested in the clinical studies were γ-secretase inhibitors (GSIs) [56, 63]. Long-term GSI treatment designed to produce moderate levels of inhibition of γ-secretase has been shown to be associated with unacceptable side effects and lack of clinical efficacy in AD [74, 75]. Side effects appear to be mechanisms based and, in many cases, can be linked to inhibition of NOTCH1 cleavage and signaling [56, 63]. Though GSIs have been repurposed for various cancers and remain under clinical investigation, development for use in AD has been discontinued. β-secretase inhibitors have also been extensively tested in the clinic and were once the major hope for a small-molecule approach to treatment or prevention of AD [76–83]; however, numerous studies in symptomatic AD or MCI showed no evidence for disease modification despite robust lowering of CSF Aβ, and numerous adverse effects that were not anticipated based on preclinical studies (reviewed in [83]). Indeed, phase 2 and 3 trials of β-secretase inhibitors showed no benefit, or were discontinued, due to futility, adverse events, or some combination of these. Unexpected adverse effects included early, mild cognitive impairment that appears to be non-progressive and reversible upon discontinuation. These adverse effects are almost certainly “on-target” and likely reflect the adverse effects of chronic and high-level inhibition of β-secretase. Though these data have renewed arguments about possible physiologic roles for Aβ [84], it is more likely that toxicity of both GSIs and β-secretase inhibitors is attributable to the net impact on signaling events mediated by the myriad of substrates each of the proteases cleave. Several small molecules referred to as γ-secretase modulators (GSMs) that selectively reduce Aβ42/43 levels and increase the levels of shorter Aβ peptides have also been developed and advanced into clinical trials [63, 85–88]. These agents showed limited target engagement in humans and had a narrow therapeutic index which was thought to be due to off-target effects.

#### What Have We Learned from These Studies?

The experience with all of these small-molecule agents targeting Aβ highlights the difficulties of an Aβ-centric approach to AD. Indeed, my colleagues and I have previously discussed in multiple reviews and perspectives the dilemma of targeting Aβ in the symptomatic phase of disease as opposed to prevention [10, 55, 57]. Given both the triggering role of Aβ aggregation and accumulation in disease and the long standing and widespread damage to the brain at the time most individuals are diagnosed with any form of cognitive impairment, inhibiting production or Aβ aggregation almost certainly requires a prophylactic approach to be initiated a decade or more before onset of symptoms. This means the drug must be very safe and must also engage target sufficiently to warrant the long testing necessary to evaluate potential for disease modification. Furthermore, from a public health perspective, the drug needs to be affordable.

### Open Questions for Aβ Production and Aggregation Inhibitors

1. *Given that these agents are only likely to have impact on disease in primary or secondary prevention is there any path forward?* β-secretase inhibitors that lowered CNS Aβ levels failed to impact clinical progression in trials in symptomatic AD. These studies, again, represented important negative data for the field, firmly supporting assertions that targeting Aβ production would require prophylactic treatment long before appearance of symptoms; however, the data showing that several β-secretase inhibitors worsened cognition and caused brain volume loss in cognitively normal at-risk individuals has currently halted further development of these agents. Though a recent perspective suggests that there may be paths to revive clinical development β-secretase inhibitors, the authors acknowledge that more data is needed and that these efforts will be challenging [83].
  Ongoing efforts to conduct prevention studies in AD are providing invaluable and ever evolving paradigms for how to conduct both primary and secondary prevention studies in Alzheimer’s disease, but the paucity of agents that meet the safe enough, sufficient targeting engagement, and rationale for disease medication criteria is worrisome [89–93]. Indeed, what seemed like a robust therapeutic pipeline for prevention studies has rapidly dwindled over the last few years.
2. *Are there any approaches to targeting Aβ safely that make sense?* From a theoretical standpoint, both GSMs and small molecule aggregation inhibitors remain attractive approaches to “safe enough” targeting of Aβ. Indeed, for GSMs, there is extensive data that support both the safety and selectivity of this approach and the potential protective action of both lowering Aβ42 levels and increasing the level of shorter Aβ peptides; however, it has been challenging to develop GSMs that are brain penetrant and that lack what appears to be off-target toxicity. Nevertheless, a few preclinical GSMs programs remain active and appear to have optimized many of the properties of the drugs [94, 95]. Given the rationale for selective targeting of Aβ42 along with the challenges of targeting β-secretase, renewed efforts to develop novel, safe GSMs and test these agents in prophylactic paradigms would make sense.
  Aβ aggregation inhibitors have always made sense conceptually, but have been plagued by issues of potency and evidence for in vivo efficacy even in the preclinical setting. Given the advances in the structural details of Aβ assemblies and mechanisms of nucleation and fibril elongation, it might be worth revisiting past efforts to try to drug Aβ aggregation [96, 97]. Obviously, it is challenging to revive efforts that were largely unsuccessful in the past, but given new data and new mechanistic insights, it may be possible to overcome limitations of previous approaches. Given prior efforts, general issues around specificity of aggregation inhibitors, and stoichiometry of small molecules typically needed to alter aggregation of proteins, new initiatives should proceed cautiously and include screens for potency and specificity early in the discovery process.
  Other efforts such as antisense oligonucleotide (ASO)-based targeting of APP or other gene therapies to target Aβ production or APP levels certainly are plausible scientific approaches, but face implementation challenges due to issues around safety and population-scale delivery [98]. If targeting Aβ showed more evidence for efficacy in symptomatic individuals, then these efforts might make more sense if they could be shown to overcome safety issues of other approaches; however, given available data, it seems that major technological challenges would need to be overcome before testing these approaches as prophylactic therapies.
3. *Can new model systems help?* Current cellular and animal models of Aβ production, clearance, and deposition are more than adequate models when used for pharmacodynamic studies that assess target engagement and effects on Aβ [99]. The informativeness of behavioral readouts in rodent models of amyloid deposition is much less certain, and it is clear that these models do not reflect the neurodegenerative symptomatic phase of AD. Though previous studies have indicated that crosstalk between Aβ and tau pathologies can be modeled in transgenic mice [100, 101], there is still a huge gap in our understanding of what factors mediate that crosstalk. Model systems that reproducibly and faithfully recapitulate the crosstalk between Aβ and tau as well as show robust neurodegenerative changes would have a huge impact on the field and likely lead to the identification of novel therapeutic strategies that could impact the course of disease in more advanced preclinical or even symptomatic stages.

## Therapeutic Approaches Targeting Tau

### Overall Rationale

Accumulation of tau as neurofibrillary tangles is a hallmark AD pathology [102]. Numerous studies link abnormal tau metabolism to neuronal dysfunction and death. Mutations in the gene (*MAPT*) encoding the tau protein that cause frontotemporal dementia with parkinsonism linked to chromosome 17 (FTDP-17 *MAPT*) demonstrate that alterations in tau are sufficient to cause neurodegeneration [103]. Genetic and animal modeling data from the study of FTDP-17 *MAPT* only directly support tau as a therapeutic target in FTDP-17 MAPT; however, given the prominence of tau pathology in the AD brain, its correlation with clinical symptoms, and experimental evidence that Aβ can drive tau pathology in vivo, tau remains an attractive theoretical target in AD [104]. It has been somewhat more challenging to identify tau therapeutics as opposed to Aβ-targeting therapies [105]. Pathologically associated tau is extensively post-translationally modified (PTM) with modifications including, but not limited to, extensive phosphorylation, acetylation, and O-glycosylation [106, 107]. Tau PTMs are altered as tau accumulates in the brain and there is evidence that modifying the activity of proteins that control the PTM can alter the normal cellular function of tau and its subcellular distribution, but also alter its aggregation [108]. Though select kinase inhibition and tau aggregation inhibitors have been the focus of previous efforts, clinical data supporting these approaches remains underwhelming. Nevertheless, new kinase inhibitor targets remain under development. Most recent efforts have focused on immunotherapies targeting tau as well efforts to alter tau though alteration in O-glycosylation. In the following paragraphs, I will discuss the status of these and open questions arising form completed and ongoing studies.

### Immunotherapies Targeting Tau in AD

#### Rationale

The notion that antibody-based therapies could target tau pathology and tau-related neurodegeneration was quite controversial following publication of initial preclinical data [109, 110]. Three possible mechanisms have been proposed to account for the apparent efficacy in preclinical settings. The first of these proposed mechanisms is that tau-targeting antibodies can bind to extracellular tau “seeds” that help to spread tau pathology between cells in the brain. Preclinic studies demonstrate that prion-like seeding of tau pathology via this extracellular seed occurs in select model systems and can be mimicked by exogenous application of aggregated tau seeds [111, 112]. Tau antibodies can intercept extracellular tau seeds and block spread of pathology by binding and neutralizing the tau seed, diverting the seed to be phagocytosed by microglial, or enhancing export from the brain. The second proposed mechanism is that tau-binding antibodies are internalized into neurons through Fc receptors [110, 113–115]. This mechanism remains controversial as the evidence for neuronal cell surface Fc receptor expression is controversial. Furthermore, it is difficult to envisage how an antibody binding to a cell surface Fc receptor might be internalized and then capable of targeting tau in the cytoplasm. A third mechanism involves possible uptake of the tau seed and bound anti-tau antibody complex by neurons or other cells and then binding of this complex to the intracellular Fc binding TRIM21 protein [116, 117]. TRIM21 binds the Fc domains of IgGs with high affinity and contains an E3-ligase domain. TRIM21 was originally shown to mediate intracellular clearance of antibody-coated viruses by catalyzing ubiquitination of antibody bound virus and degradation via the ubiquitin proteasome system and similar data exists that TRIM21 can play a role in degrading internalized tau aggregates [116, 117]. Notably, several studies show that single domain variable fragments lacking Fc domain can modulate tau pathology in vivo; such data would suggest that Fc-mediated mechanisms are not responsible for efficy of tau immunotherapies [118–120]. Though most therapeutic approaches have focused on using monoclonal tau antibodies, active immunization strategies have also been pursued.

#### Clinical Studies

Multiple monoclonal tau antibodies have been tested in human studies [121–134]. In many cases, the antibodies were tested in mild AD and in separate trials in the primary tauopathy progressive supranuclear palsy (PSP). Many of these trials have been discontinued as there has been little evidence for efficacy. Several active vaccine strategies remain under clinical development, though little data besides induction of antibody titer and safety have emerged [135–138]. In many of the passive immunotherapy studies, there was evidence for entry of the antibody into the CNS and evidence that the antibody was able to bind the amino terminal fragment of tau that is normally present in CSF and brain extracellular fluid. Clinical evidence for disease modification with these antibodies has been negative to date.

### Open Questions for Tau Immunotherapies

1. *Does Prion-like spread of tau occur in the human brain?* Much of the enthusiasm to pursue tau immunotherapy is based on the preclinical data that tau can spread in a prion-like fashion. Prion-like spread of tau *in humans* remains a hypothetical event [139]. If it does not occur, then it would seem that efforts to target extracellular tau have a very low probability of success. Unfortunately, showing that an antibody therapy blocked tau spread in humans is one of the few direct tests of this hypothesis, but for reasons described in the answer to the next question, it is not clear whether the binding properties of any antibody tested in humans are sufficient to have tested the hypothesis.
2. *Are any anti-tau antibodies tested in humans targeting the right domain of tau and of high enough affinity to engage the tau “seed,” even if it is present?* Studies of extracellular tau in human spinal fluid show that most tau present in the CSF lacks the carboxyl terminal microtubule domains that appear to be the core amyloidogenic region of tau [107]. Thus, most tau in the CSF and interstitial fluid is thought to be incapable of seeding tau aggregation. In these same studies, a much lower level of tau containing the microtubule binding domains was detected. Indeed, these studies indicate that maximal levels of extracellular tau that could theoretically serve as a seed were approximately 1–2 pM. Further studies of human AD CSF show that species capable of seeding may be present, but are present at extremely low levels [140]. From target engagement and pharmacologic perspectives, these data suggest that in order for a tau seed to be engaged by an antibody within the brain, the tau-targeting needs to have extremely high affinity, or avidity, and be capable of targeting the carboxyl terminal domains of tau that are present in the putative seeding competent species. Indeed, antibodies that bind the mid-domain or amino terminal regions of tau would likely bind preferentially to the much more abundant amino-terminal species present in CSF and brain interstitial fluid. Given that the maximal brain levels of any peripherally delivered antibody are predicted to be in the 1–5-nM range, it is hard to envision how low pM, or even sub pM concentrations, of a tau seed could be effectively engaged by an antibody unless the antibody was selective for the seed and had low pM or sub-pM avidity. Based on available data, it is not clear if any antibody tested to date has such properties.
  Pathologically associated tau is extensively post-translationally modified (PTM); thus, there are a plethora of epitopes and disease-associated PTM epitopes that theoretically could be targeted using an immunotherapy [106, 107, 141]. It remains possible that a specific epitope uniquely defines naturally existing extracellular tau seeds. Antibodies with exquisite specificity and affinity for this theoretical seed could be the magic bullets needed to target tauopathy in AD, but our current knowledge about whether such tau seeds really do exist and whether they contain such unique epitopes is insufficient to frame efforts to design better tau antibodies. Nevertheless, as more information emerges, the field should think critically about whether that data informs on the desired properties of a second-generation tau-targeting immunotherapy.
3. *Could an active tau immunotherapy produce a sufficient immune response to result in efficacy*? Given the likely requirement of the need to induce a very high affinity tau “seed” targeting humoral immune response, it would seem very challenging to reproducibly generate such a response using an active vaccine approach. Again, as vaccines could elicit T-cell-mediated responses to a tau epitope, it remains plausible that such responses could play a beneficial role, but as with Aβ vaccines, the likelihood is that such responses would probably do more harm than good.
4. *Is it possible to target intracellular tau using an immunotherapeutic approach?* Tau pathology is primarily intracellular. Preclinical studies have shown that intracellular tau targeting recombinant antibody fragments delivered with recombinant adenoassociated viral vectors (intrabodies) can have modest impact on tau pathology and tau-induced neurodegenertive phenotypes in preclinical models [120, 142]; however, given the widespread distribution of tau pathology in the brain, advances in transgene delivery using viral vectors or other methodologies are needed in order to obtain sufficient “coverage” to actually evaluate potential for disease modification. Future advances in transgene delivery and antibody engineering could eventually overcome these current limitations, but it is not easy to see how the current obstacles to targeting tau with an intrabody-like approach can be overcome in the near term.
5. *Are preclinical models of tau-pathology reliable and informative enough to guide human therapeutic development?* Preclinical models of tau pathology are really modeling FTDP-17 tauopathies and not AD tauopathy. Moreover, many widely used transgenic lines show variable phenotypes [143] and more recently tau-induced neurodegeneration in the commonly used Tg4510 line has been shown to be influenced by transgene insertion events [144]. Seeded models including seeding of endogenous mouse tau have been developed and could be useful preclinic models, but, again, are quite artificial with respect to how they are generated [145]. More generally, the interpretation of efficacy in these models is often based solely on reduction of pathological tau and not on a neurodegenertive phenotype. Furthermore, robust reductions of tau pathology have not been associated with major impact on neurodegenertive phenotypes in select tau mouse models.
6. *Do we understand tau pathobiology well enough to target tau successfully?* More generally, the issue of how tau pathology is associated with neurodegeneration remains poorly understood [108, 146, 147]. Provocative studies demonstrate that tangle bearing neurons remain functional at least for some period of time [148]. If neurofibrillary tangles themselves were acute toxins, then most cells bearing them would die quickly. This acute death is clearly not the case as most tangle bearing cells are clearly intact. Tau PET ligands would also not be very useful biomarkers if tau inclusion pathology did not increase over time in the human AD brain. Our limited understanding of both tangle formation in the setting of AD and the role that tangle formation or tau dysfunction plays in neurodegeneration remains a major knowledge gap and clearly limits our ability to design tau-targeting therapies that are more likely to show clinical efficacy. Recent studies also highlight that fibrillar tau inclusions form rapidly and turnover with an appreciable half-life [149, 150]. Such data suggests that cells adapt at least in the short term to tau inclusion formation. Though the concept of reducing tau pathology is attractive, it is possible that tau inclusion formation is in fact an adaptive, protective response, and that efforts to reduce it may have unintended negative impacts.

### Small Molecule and ASO Approaches to Targeting Tau

#### Rationale

Most small molecules targeting tau are designed to directly or indirectly alter its aggregation. The notable exception being ongoing development of select microtubule stabilizing agents that designed to compensate for loss of microtubule binding by hyperphosphorylated or aggregated tau. Modulators of tau PTMs, tau-chaperones, and tau aggregation inhibitors have all advanced to clinical trials, but most programs have been discontinued. In the paragraph below, I will briefly highlight the rationale for these approaches with a focus on approaches that remain in clinical development.

As noted above, tau has extensive PTMs including phosphorylation, acetylation, and O-linked glycosylation [106, 107, 141]. These various PTMs have been implicated as possible sites for therapy as altering the PTMs appears to regulate tau aggregation and toxicity in preclinical models [151]. One of the challenges with targeting tau PTMs with small molecules is that phosphorylation, acetylation, and glycosylation are all reversible dynamic modifications and multiple phosphatases, kinases, acetylases, deacetylases, glycosylases, and glycoside hydrolases regulate the extent of tau PTM [106, 107, 141]. The action of these enzymes is not specific to tau, but regulate PTMs on many proteins and through this alter many cellular processes; thus, small-molecules targeting the enzymes that regulate tau PTM will impact numerous proteins and cellular functions. Previous efforts to target tau by inhibition of GSK3β have been largely discontinued [152] and most current efforts have focused on inhibition of O-GlcNAcase, the glycoside hydrolase enzyme that removes O-linked N-acetylglucosamine (N-GlcNAc) from proteins. N-GlcNAcylation of the microtubule-associated protein tau reduces its propensity to form toxic aggregates and is thought to compete for phosphorylation at those residues [153]. Indeed, increasing tau glycosylation is proposed to stabilize tau in a non-toxic form and several O-GlcNAcase inhibitors including Thiamet G have shown beneficial effects in preclinical models of tauopathy [154–162].

Many chaperone proteins including, but not limited to, HSP90, CHIP, and FKBP51 have been implicated as tau chaperones [163–165]. These chaperones can, in experimental systems, regulate tau aggregation and accumulation; there are no active clinical studies using drugs that target these chaperones in AD, though preclinical efforts to try to harness chaperones that regulate tau proteostasis remains active. At least three clinical programs evaluating tau aggregation inhibitors remain active. Two of these trials are assessing methylene blue in different formulations and though methylene blue is touted as an aggregation inhibitor, it may have more complex effects on chaperone systems [166, 167]. Another, Anle138b, is reported to be a more general aggregation inhibitor and is also being evaluated in the setting of Parkinson’s disease as an α-synuclein aggregation inhibitor. ASOs targeting tau are designed to simply reduce tau levels and slow tau aggregation. Preclinical data supports tau ASOs as possible way to reduce tau aggregation and slow tau-induced neurodegeneration [168].

#### Clinical Studies

Most small molecule approaches targeting tau are either in early stage clinical trials, have been discontinued, or remain active despite underwhelming clinical data. Three O-GlcNAcase inhibitors are in phase I or II trials with insufficient data at this point to discuss potential efficacy. Methylene Blue remains in trials in a new formulation with reports of some impact on cognition. Though because of challenges with blinding, trial design, and methodology, many in the field are skeptical of the clinical benefit. At least one microtubule stabilizer remains in clinical development for PSP, but other stabilizer programs have been discontinued. BIIB080, a tau targeting ASO developed by Biogen and Ionis, was shown to be well tolerated in phase 1, but data on efficacy is not avaible.

### Open Questions for Tau Targeting Small Molecules and ASOs

1. *Is tau proteostasis so complex or so poorly understood that current efforts are likely to be futile?* Like many intracellular neurodegenerative proteinopathies, there are many aspects of tauopathy that are poorly understood. Despite robust efforts to alter tau aggregation and accumulation, even the best preclinical studies show relatively modest impact on tau aggregation and tau-associated neurodegenertive phenotypes. Given these rather modest impacts on pathology and neurodegeneration, we should probably temper our expectations regarding likelihood of impact in clinical AD.
2. *Are studies of tau-targeting agents in primary tauopathies such as FTDP-17 or PSP reasonable surrogates for studies in AD?* This clinical development strategy has been used without success for a number of tau therapeutics; however, it is reasonable to ask whether PSP or FTDP-17 are truly good phenocopies of the tauopathy in AD. Given emerging data that different conformers of tau may predominate in different tauopathies [169, 170], we should be cognizant that the different tauopathies may be distinct disorders and success or failure of a therapeutic in one disease may not be predictive of its success or failure in another.
3. *Is symptomatic disease too late?* Given that tau pathology correlates more closely with signs of neurodegeneration and clinical symptomatology, it is postulated that successful targeting of tau may be more effective in symptomatic AD; however, most preclinical data on tau therapies shows modest signs of efficacy when the therapy is initiated prior to pathology or when minimal pathology is present. Again, lack of robust effects on late-stage preclinical models of tauopathy would suggest that we should temper expectations of major benefits in symptomatic stages of disease [120]. Though amyloid pathology has largely plateaued when AD symptoms appear, tau pathology continues to progress [52, 171–173]. These relationships mean that it may be possible to target tau in the symptomatic phase and observe both a biomarker and clinical effect; however, given relatively modest impacts of most tau therapies in preclinical studies, it is likely agents with a higher degree of target engagement and disease-modifying potential will be needed in order to impact the course of disease in the neurodegenerative and symptomatic phase.
4. *Will tau PET ligands or other biomarkers serve as proxies for clinical efficacy?* Demonstration of highly significant clinical benefit in phase 2 studies in AD is very challenging. Thus, having biomarkers that serve as potential proxies for clinical efficacy and provide evidence for target modification is invaluable. Tau PET ligand signals appear to track well with clinical progression, and it is certainly hoped that they can serve as an early predictive marker of efficacy [174, 175]. In addition, given lessons learned from trials targeting Aβ, it would seem we should more systematically enroll trial participants in autopsy programs so that we can correlate effects on pathology with changes observed in imaging studies. Of note here, is that measures of tau or phospho-tau in CSF and blood are linked more tightly to amyloid deposition than tau-pathology per se [176–178]. Some recent reports do suggest that CSF and plasma tau levels continue to increase as disease progresses, but the biological correlates of these associations remain enigmatic. A recent study in mice shows that increases in extracellular tau appear to be a response to both Aβ amyloid and Danish familial dementia amyloid [179]. Notably, most extracellular tau is an amino terminal fragment of full-length tau that lacks the carboxyl terminus. Such data points to crosstalk between amyloid and tau pathologies that can be independent of tau pathology. The lack of mechanistic insight into this aspect of crosstalk between amyloid and tau is, again, a gap in our understanding that the field should address.

## Therapies Targeting Immune Activation States in AD

### Rationale

Neuroinflammation has been a long-standing, but until more recently ill-defined, target for AD and many other neurodegenerative diseases [180]. Genetic data that firmly links proteins that regulate microglial and other innate immune responses to AD risk has recently elevated the interest in novel developing immune modulators as disease-modifying agents [181, 182]. Genetic, pathologic, and experimental modeling data now strongly supports the notion that activation of innate immune signaling pathways in the brain confers protection from AD and that inhibition of these pathways may confer risk (reviewed in [180]). Such data suggests that therapeutics designed to activate the immune systems are likely to provide benefit in AD and directly contradict the long-standing dogma that inhibition of inflammatory and innate immune pathways would be beneficial.

I have recently extensively reviewed how the emerging genetic data and previous pathological, modeling, and epidemiological data lead to fundamentally distinct interpretations of how to manipulate the immune system for benefit in AD [180]. As there is yet little consensus in the field regarding directionality, both immune activating and immune inhibitory approaches are being developed and tested clinically. Indeed, a CSF1R antagonist [183], TNFα antagonists [184, 185], an NLRPL3 antagonist [186, 187], a p38 MAPK inhibitor [188], Chromyln in combination with ibuprofen [189], lenadolidomide [190], and other approaches are being pursued conceptually as immune inhibitory strategies in AD. In contrast, GM-CSF (a CSFR1 activating ligand) [191, 192], TREM2 agonists (both small molecule and antibody-based approaches) [193], and a CD33/Siglec3 antagonists [194] (antibody) are being developed as immune activating approaches for AD. Additional therapeutics with less clear-cut actions are also being evaluated. Given the large number of agents and the lack of any truly positive data from clinical studies, I will not summarize the clinical data and simply highlight the most pressing open questions.

### Open Questions for Immune Modulation in AD

1. *Will immune manipulation in any direction be safe enough in an elderly population to conduct long-term studies in AD?* Therapies that modulate the immune system often are accompanied by untoward side effects. Such side effects are often better tolerated in younger individuals or when the therapy is intermittently used. Clinical experience of immune activation strategies in cancer highlights the many untoward side effects of these therapies. It is likely that immune therapies used in AD that are sufficient to modulate immune activation states in the brain will be accompanied by some peripheral side effects. Given the high bar for safety and lack of negative effects of the therapy on functional and cognitive outcomes, it may be challenging to find a therapeutic window which permits sufficient target engagement without inducing significant side effects.
2. *Do we have good enough biomarkers to assess target engagement and modulation of immune activation states in the brain?* Although PET ligands that assess microgliosis are utilized, the signal to noise ratio of these ligands based on binding to TSPO is relatively small especially in the elderly [195]. Furthermore, how such ligands inform on immune activation states is unclear. CSF fluid biomarkers of brain inflammation are also not well validated [196]. Unless clean robust data showing clinical efficacy emerges in early phase studies, advances in biomarkers that assess brain immune activation states will likely be needed to ensure appropriate go-no go decisions are made during clinical development of these various agents.
3. *If manipulation of many immune targets seems to have differential impacts on amyloid and tau pathology and variable impacts on synaptic and behavioral functions in preclinical models might we do more harm than good?* Not all immune targeting therapies are evaluated in both amyloid and tau models before they enter clinical studies. Given numerous published examples of disparate impacts of an immune manipulation on tau, amyloid, and neurodegeneration in preclinical studies, it may be that an immune targeting therapy does more harm than good (reviewed in [180]). Indeed, both scientists and regulatory bodies probably should insist on more thorough preclinical assessments of AD immune therapies, or at least ensure that both amyloid and tau pathologies are assessed during the course of the trial.
4. *When during disease progression should an immune modulatory drug be tested?* Immune manipulations may have beneficial or harmful consequences on brain function if they have different impacts on amyloid, tau, and neuronal or brain circuit function. The possible disease state dependence of efficacy raises many issues with respect to clinical testing of immune modulators in AD. Clinical testing with these agents is largely conducted in mild AD patients and that will likely be the initial intent to treat population unless we have more refined biomarkers of innate immune activation states in the brain. As these therapies may impact amyloid, tau, and neurodegeneration, it is imperative that trials monitor these pathologies using avaible biomarkers.

## Lessons Learned from Two Decades of Disease-Modifying Trials

The first lesson is that disease modification in AD is hard. Given the slow and variable rates of symptom progression, it is difficult, though possible, to measure clinically, and only recently do we have biomarkers that can enable tracking of progression in the long prodromal silent phase of the disease as the pathology develops. Large-scale trials designed to assess impacts on cognitive and functional decline can assess clinical impacts, but these trials remain expensive and are lengthy trials to conduct. Though the recent FDA approval of Aduhelm indicates a willingness to potentially use a biomarker of pathology as a surrogate for clinical efficacy, the long-term outcomes of a biomarker-based approval may not always prove to achieve a positive benefit to risk impact. Perhaps with improvements in the power of biomarkers to predict future cognitive trajectories, it will be possible to assess efficacy of a potentially disease-modifying agent in the presymptomatic phase of disease in relatively small cohorts [197, 198].

The second lesson is that use of biomarkers to both identify and stratify intent to treat populations with dementia or preclinical stages of dementia and to track impact on underlying pathology progression is almost requisite to conduct a rigorous clinical study. Routine use of biomarkers, especially imaging modalities, adds significant costs and participant burden to the trial. Nevertheless, without these biomarkers, we are in many ways blind to impact on underlying pathologies and possible unanticipated adverse effects. Furthermore, if one is targeting an AD pathology and the trial includes participants without AD, not only is the trial potentially confounded, but ethically we are exposing individuals who are highly unlikely to benefit from the trial to an unnecessary risk.

A third lesson is that when conducting disease-modifying trials in AD, we should try our best to enroll participants in brain autopsy donation programs. Most AD trials enroll elderly individuals and obtaining and studying their brain following an intervention might provide key insights into the efficacy or adverse effects of a given therapy. If this had been standard of practice for the last decade, then we would likely have more insight into the relationship between amyloid signal reductions, actual effects on amyloid pathology, and some insight into the biological underpinnings of ARIA-E.

A fourth lesson is that we really do need to do better on the practices of good therapeutic development practices. In the absence of an unambiguous beneficial clinical signal, compelling evidence for target engement and supporting evidence for possible disease modification should be ascertained during early phase trials. Without such data, large-scale phase 3 studies have very low probabilities of success and are not likely to inform the field as to how to move forward.

A fifth lesson is that we should do our best to test agents at the time in disease progression when they are more likely to have an impact on disease. The concept of testing therapeutics at the right time was a major focus of recent perspective written by me and several colleagues [55]. Indeed, the current debate over the possible efficacy of Aduhelm in symptomatic AD partially deflects us from addressing the critical unanswered questions about Aduhelm and other Aβ targeting antibodies that appear to engage and modify the target pathology in the brain. That question is as follows: “Might these antibodies work in primary or secondary prevention studies?” If Aduhelm or another antibody truly prevented or substantially reduced amyloid deposition during the long prodromal silent phase of AD, then this or a similar approach may ultimately be how we prevent AD. Alternatively, if it did not alter the clinical appearance of AD despite impacting amyloid deposition, then we would have to critically rethink the role of amyloid in AD. Unfortunately, Aduhelm was never included in any of the large-scale prevention studies that have been underway, and we will have to wait many more years before we have an answer to the critical question of whether targeting amyloid in the prodromal phase of disease impacts subsequent development of AD.

## Conclusions—Challenges for the Field

Building off the first lesson that disease modification is hard, I will conclude with several challenges to the field. The first is that a patient suffering from AD does not really care about disease modification; they want their symptoms to improve or disease progression to be dramatically halted. A slight slowing of decline in cognition and functional ability (20% per year) is really not something the patient or caregiver will be able to discern, at least in the short term. Thus, we need to think about ways to dramatically improve cognitive and functional status in the symptomatic phase or, if we are shooting for disease modification, aim for a much larger impact on functional decline.

In order to achieve larger impacts in the clinical phase of AD, we likely need to revisit development of symptomatic therapies designed to directly improve cognition and function. Many targets that were once thought to be undruggable can now be successfully targeted and new targets that might improve cognitive function independent of pathology have been identified. Though development of cognitive enhancing agents has not been completely ignored and a few promising therapies are in clinical development, the resources behind efforts to develop symptomatic therapies are miniscule in comparison to the resources supporting the largely failed efforts to develop disease-modifying agents.

As noted above, another key challenge is to really try to align clinical testing of agents that are designed to be disease modifying in the stage of disease that might provide the largest clinical signal. Many of the scientific challenges to conducting such trials have now been largely overcome; we can identify individuals with underlying AD pathologies and track progression of those pathologies long before clinical symptoms emerge. We can also use genetics and biomarkers to assess individuals at risk for disease even before radiologic evidence of pathology emerges; however, testing therapies in prevention remains challenging, as there many financial and practical disincentives to conducting such studies. As a society, we need to think how to incentivize the private sector to conduct or participate in prevention studies in a way that produces an affordable public health solution to prevention of AD. If, for example, CMS does pay for the cost of Aduhelm in ongoing clinical trials, maybe there can be a more codified public–private cost-sharing in extended trial models that could help support the long-term studies of therapies designed for AD prevention. This cost-sharing could reduce the financial risk for a private sector and help them to engage in the types of trials that are likely to yield the biggest public health impact. Certainly, current prevention initiatives illustrate the power of these partnerships [90, 92, 199], but a more standardized regulatory path with appropriate financial models that is jointly developed with the many public and private stakeholders might ensure that these partnerships are lasting and not undermined by changes in the regulatory and payer landscape.

The bar for safety is also very high for any therapeutic used as a prevention agent. I have previously suggested such a drug needs to be at least as safe as a statin [10]. Given the toxicities observed with most anti-Aβ therapies that have human data supporting target engagement, the field needs to grapple with the possibility that a true prevention study that tests the core postulate of the amyloid cascade hypothesis that preventing amyloid position will prevent AD is unlikely to occur in the near term unless we identify safer approaches.

A final challenge is that we likely need to think about disease modification in symptomatic AD a little differently in order to achieve the larger impact needed. AD is an incredibly complex disorder and by the time symptoms manifest almost all cell types within the brain have been altered and pathology building for over two decades. Thus, the notion that we can significantly impact decline in symptomatic AD with a single agent is likely wishful thinking. We need to approach symptomatic AD more like organ failure and develop cocktails of agents that both target underlying pathologies, impact cognitive function, and have some ability to regenerate and repair the damage. Such combinatorial therapies will be challenging to develop and must utilize creative non-dogmatic approaches based in rigorous translational science. Such efforts will likely yield more failure than successes, but unless we rigorously and systematically try to develop such a combinatorial approach, we cannot succeed. Indeed, we are likely at least a decade or two away from identifying safe highly effective prophylactic interventions for AD and universally deploying them. Thus, symptomatic AD will continue to be a prevalent and growing problem and we must take disruptive high-risk approaches to improving lives of those suffering with it, or at risk for developing it, in the future.

## Supplementary Information

Below is the link to the electronic supplementary material.

Supplementary file1 (PDF 508 kb)
